# Sentiment Analysis of Online Reviews for Selective Serotonin Reuptake Inhibitors and Serotonin–Norepinephrine Reuptake Inhibitors

**DOI:** 10.3390/pharmacy9010027

**Published:** 2021-01-23

**Authors:** Chad Compagner, Corey Lester, Michael Dorsch

**Affiliations:** College of Pharmacy, University of Michigan, Ann Arbor, MI 48109, USA; chadcomp@med.umich.edu (C.C.); mdorsch@med.umich.edu (M.D.)

**Keywords:** sentiment, antidepressant, depression, emotion, SSRI, SNRI

## Abstract

Background: Depression affects millions worldwide, with drug therapy being the mainstay treatment. A variety of factors, including personal reviews, are involved in the success or failure of medication therapy. This study looked to characterize the sentiment of online medication reviews of Selective Serotonin Reuptake Inhibitors (SSRIs) and Serotonin–Norepinephrine Reuptake Inhibitor (SNRIs) used to treat depression. Methods: The publicly available data source used was the Drug Review Dataset from the University of California Irvine Machine Learning Repository. The dataset contained the following variables: ID, drug name, condition, review, rating, date, and usefulness count. This study utilized a sentiment analysis of free-text, online reviews via the sentimentr package. A Mann–Whitney U test was used for comparisons. Results: The average sentiment was higher in SSRIs compared to SNRIs (0.065 vs. 0.005, *p* < 0.001). The average sentiment was also found to be higher in high-rated reviews than in low-rated reviews (0.169 vs. −0.367, *p* < 0.001). Ratings were similar in the high-rated SSRI group and high-rated SNRI group (9.19 vs. 9.19). Conclusions: This study supports the use of sentiment analysis using the AFINN lexicon, as the lexicon showed a difference in sentiment between high-rated reviews from low-rated reviews. This study also found that SNRIs have more negative sentiment and lower-rated reviews than SSRIs.

## 1. Introduction

The World Health Organization reports that over 264 million people are affected by depression [1]. The 2018 National Survey on Drug Use and Health in the United States found that 3.7 million people aged 12–25 were being treated for depression, with 8.1 million in the same age group experiencing a major depressive episode in the last 12 months [2]. In adults age 26 or older in the United States, 13.1 million people suffered a major depressive episode in the last 12 months [2]. Overall, depression is a disorder that affects many people worldwide, as well as in the United States. According to the American Psychiatry Association, first-line therapy for depression includes pharmacologic therapy, such as a Selective Serotonin Reuptake Inhibitor (SSRI) or Serotonin–Norepinephrine Reuptake Inhibitor (SNRI) [3].

Previous studies have shown that patients are often nonadherent to their antidepressant regimen due to a variety of reasons [4]. One reason for potential nonadherence is reports of another patient’s experience. A Pew Research Center study found that 48% of insured internet users sought out online information about their treatment, and 40% posted their stories about their own experiences [5]. Another study found that patients were more likely to choose varenicline after being exposed to positive reviews of the medications [6]. Positive or negative patient experiences can influence adherence, thereby affecting treatment success. Reviews of medications can be found on sites such as Drugs.com^®^ or WebMD^®^. These websites provide information to individuals with only a web search on the internet.

Limited studies have sought to better understand the reviews that currently exist on the internet. One study performed a sentiment analysis, using the Valence Aware Dictionary and Sentiment Reasoner on approximately 13,000 websites related to psychotropic medications [7]. The authors found that the sentiment varied significantly between classes of psychotropic medications, as well as within a single class. They did not find a consistent trend in their analysis. Their analysis focused on websites found from a web search which could include manufacturer websites. The study also did not focus on personal reviews of the drugs that would likely have different content than would be found on a manufacturer’s informational website. Our study looked to improve previous work by looking at the sentiment of patient reviews, to gain an understanding of their sentiment. Our study also focused on two commonly prescribed classes of antidepressants for their most common indication instead of broad classes of psychotropic agents for any indication.

The objective of this study was to evaluate the sentiment of the reviews of SSRIs and SNRIs used to treat depression. A secondary goal of this study was to determine if there is any difference in the patient rating of the medication between SSRIs and SNRIs in the treatment of depression. By analyzing the sentiment of unsolicited reviews of SSRIs and SNRIs pulled from Drugs.com^®^, this study provides insight into the differences in reviews between SSRIs and SNRIs, in both sentiment and rating of their experience and the sentiment of their review.

## 2. Materials and Methods

**Figure 1:**
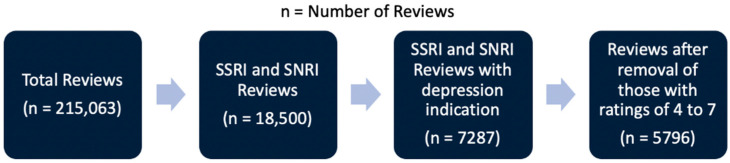
Summary of filtering and word processing. Starting from the left, total reviews were filtered to Selective Serotonin Reuptake Inhibitor (SSRI) and Serotonin–Norepinephrine Reuptake Inhibitor (SNRI) reviews. From the SSRI and SNRI reviews, the reviews were standardized and represented as individual word counts, instead of the number of reviews.

This study was a retrospective analysis of the Drugs.com^®^ dataset from the University of California Irvine Machine Learning Repository [8,9]. The dataset contained 215,063 unsolicited drug reviews pulled from Drugs.com^®^. This dataset was created by crawling Drugs.com^®^, to gather all the reviews and associated data. This study used a subset of 5796 reviews. The dataset contained the following variables: ID, drug name, condition, review, rating, date, and useful count. ID identified each review. Drug name was a nominal variable designating the medication. Condition denoted the condition that was being treated. The review was a free-text review of the medication. The rating was a value between 1 and 10 of the therapy’s effectiveness. All reviews related to a Selective Serotonin Reuptake Inhibitor (SSRI) or Serotonin–Norepinephrine Reuptake Inhibitor (SNRI) were included, which narrowed the number of reviews to 18,500. Drug names were first linked to a class (SSRI or SNRI), using a string match between the data frame containing the drug name and class and the data frame with the reviews. The following SSRIs were included: sertraline, citalopram, escitalopram, fluoxetine, fluvoxamine, and paroxetine. The following SNRIs were included: venlafaxine, desvenlafaxine, duloxetine, milnacipran, and levomilnacipran. Figure 1 shows a summary of data filtering.

### 2.1. Text Preprocessing

To look into the sentiment of the reviews, reviews were first processed to a standard form. The reviews were standardized, using the tidytext package in R, to transform the reviews into all lowercase [10]. The filtering of the data was completed by using the dplyr package [11]. The data were filtered further by including only reviews for SSRIs and SNRIs. Once filtered to SSRIs and SNRIs, the reviews were further filtered to those who reported using it for depression. Once filtered to depression, reviews with intermediate ratings, ratings of 4 to 7, were removed.

### 2.2. Sentiment

The syuzhet packages contained multiple lexicons that can be used to analyze sentiment [12]. The AFINN lexicon rated the sentiment of 3374 unique words from −5, the most negative, to 5, the most positive [13]. The AFINN lexicon is a general-purpose lexicon capable of being applied in many settings. The following terms were removed from the AFINN lexicon, as they were represented in the valence shifter table: certain, huge, kind of, no, severe, severely, significant, and true. The BING lexicon, created by Minqing Hu and Bing Liu, was considered as an alternative lexicon. The AFINN was used for the increased granularity. In a sensitivity analysis, the AFINN lexicon was better able to differentiate high- and low-rated reviews (Appendix A). The individual reviews were normalized for the length of review by creating an average sentiment per review. The average sentiment, using the sentimentr package, considers valence shifters such as account negators, amplifiers, de-amplifiers, and adversative conjunctions, to determine the sentiment at the sentence level [14]. Sentimentr looks at five words before and two words after a sentiment word for valence shifters. If a valence shifter is found, it adjusts the sentiment value accordingly. For amplifiers and de-amplifiers, the sentiment value was multiplied by 1.8. For negators, the sentiment value was multiplied by −1. For adversative conjunctions, the sentiment value was multiplied by 1.25 if it occurred before the sentiment word or by −1.25 if it occurred after the sentiment word.

Numerical ratings were split into high/low categorical ratings. The high-rated reviews were ones that were rated as 8 or higher. The low-rated reviews were ones that had a rating of 3 or lower. From there, the average sentiment was compared between high-rated and low-rated reviews, as well as between SSRIs and SNRIs. The ggplot2 package was utilized to create visual comparisons of the classes and ratings [10].

### 2.3. Analysis

First, this study compared the ratings between the two classes of antidepressants. It was hypothesized that there would be no difference in ratings between SSRIs and SNRIs. Both of these medications are indicated as first-line therapy for the treatment of depression. A few studies suggested that SSRIs are prescribed more often as the first medication used to treat depression [11,15]. It was not expected that there would be a significant difference in patient experience, and, thus, no difference in ratings was expected.

Second, this study looked to identify the difference in average sentiment between high-rated and low-rated reviews. High-rated reviews were hypothesized to have a higher average sentiment, compared to low-rated reviews, because those with positive experiences were expected to rate the medication more highly and use positive words to describe the experience.

Finally, this study compared the average sentiment between SSRIs and SNRIs. No difference in averaged sentiment was expected, as the medications are both commonly used, with similar rates of side effects. A difference in average sentiment between SSRI reviews and SNRI reviews is likely due to a difference in side-effect exposure.

These hypotheses were tested by using a Mann–Whitney U test. The Mann–Whitney U test compares random samples of nonparametric data, to determine if a difference exists between the groups. There were more reviews at the extremes of rating. Rating and sentiment were not independent of each other. For these reasons, the Mann–Whitney U was used to test for differences.

## 3. Results

**Figure 2:**
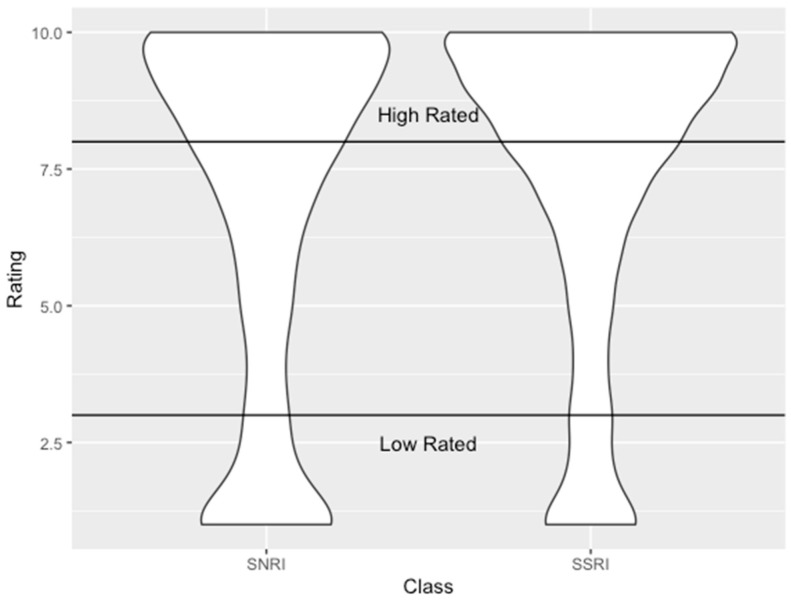
This figure shows a violin plot showing the distribution and grouping of ratings between the classes. For SSRIs, 2418 reviews fell into the high-rated group, and 621 reviews fell in the low-rated group. For SNRIs, 1946 reviews fell into the high-rated group, and 811 reviews fell in the low-rated group. Most reviews fell in the high-rated section, with the fewest reviews occurring in the middle of the ratings.

Across all medications that were analyzed, a total of 5796 were reviewed. Table 1 shows the breakdown of the ratings, word count, usefulness count, and date range. The reviews were split equally with 3039 SSRI reviews and 2757 SNRI reviews. SNRI reviews were similar in length to SSRI reviews (91.1 vs. 90.9). The ratings had a similar variation between the two groups. Both SSRIs and SNRIs have an average rating of 9.19 in the high-rated group; however, in the low-rated group, SSRIs had a higher average rating by 0.13 out of 10 when compared to SNRIs (1.67 vs. 1.54). Figure 2 shows the distribution of ratings in both SSRIs and SNRIs. Of note, SNRIs had a higher proportion of low rating reviews, compared to that of SSRIs. SNRIs had a higher proportion of reviews categorized as low-rated, compared to SSRIs (29.4% vs. 20.4%).

**Figure 3:**
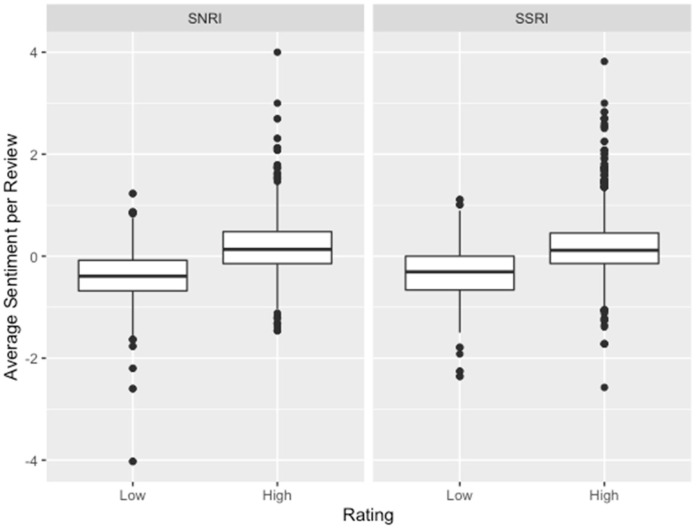
This figure shows the distribution of average sentiment between classes and ratings. High-rated reviews had higher sentiment on average. High-rated SSRI reviews had an average sentiment of 0.164, compared to 0.175 for high-rated SNRI reviews. Low-rated SSRI reviews had an average sentiment of −0.320, compared to −0.403 for low-rated SNRI reviews.

Each class of medications had reviews with negative and positive average sentiment, based on the AFINN lexicon created by Finn Årup Nielsen. For SSRIs, the average sentiment for all reviews was 0.065, with a standard deviation of 0.566. SNRIs had an average sentiment for all reviews of 0.005, with a standard deviation of 0.585. SSRIs were significantly higher by 0.06 than SNRIs (*p* < 0.001). Figure 3 shows the distribution of average sentiment between SSRIs and SNRIs. Overall, 2987 reviews had an average sentiment greater than 0, and 2649 reviews had an average sentiment less than 0.

High-rated reviews had an average sentiment of 0.169 ± 0.533, compared to low-rated reviews, which had an average sentiment of −0.367 ± 0.510. There was a significant difference in the average sentiment between those who rated the medication high, compared to those who rated it low (*p* < 0.001). Figure 3 shows the distribution of average sentiment between the rating groups. High-rated reviews had a higher average sentiment that was 0.536 higher than low-rated reviews.

An analysis of variance was performed with and without an interaction term. Without the interaction term, only the rating variable made a significant contribution to the model. This suggests that only rating grouping was driving the difference in average sentiment. With the interaction term, all terms were found to be significant. A lower average sentiment in the high-rated group for SSRIs was suggested, as compared to SNRIs in the high-rated group (*p* = 0.004). The results of the model can be seen in Table 2 and Table 3. Figure 3 shows the average sentiment distribution split between the class of medication and the rating group.

## 4. Discussion

This study analyzed 5796 reviews from the University of California Irvine Machine Learning Repository Drugs.com^®^ dataset. The sentiment of the reviews was compared between differences in rating and class. This study looked to provide greater insight between reviews and patient experience.

SSRIs and SNRIs had a similar number of reviews for the use of depression present in the dataset. Of note, the ratings for SSRIs were higher on average than those of SNRIs. Based on the American Psychiatry Association guidelines, SSRIs and SNRIs are both appropriate choices for the initial treatment of depression [3]. However, one study looked into the treatment patterns of patients from 2015 to 2019, using nationwide databases and found that between 36.3% and 57.5% of patients were treated first-line with an SSRI [16]. Another study found that nearly 89% of patients received an SSRI as initial therapy for the treatment of depression [17]. Based on these studies, patients who receive SNRIs are likely receiving it after failing an SSRI or other medication. Patients who had failed other antidepressant medications are more likely to have treatment-resistant depression, which could be skewing the reviews, as patients who receive an SNRI are at a higher risk to fail another medication. A study published in the American Journal of Psychiatry found that the percentage of people who achieved remission decreased from 36% in treatment-naïve to 30.8% after failing one medication [18]. They noted a further decreased remission rate to 13.7% after failing two treatment modalities [18]. In this way, the prescribing patterns present a likely explanation for the difference in rating and sentiment between the two medication classes. Another potential explanation of the differences that were observed in this study could be variations in the side-effect profiles between SSRIs and SNRIs. However, a systematic review of antidepressant use in the geriatric population found minimal evidence of a difference between the two classes [15]. This study suggests that both SSRIs and SNRIs led to similar rates of treatment withdrawal, based on side effects [15]. Overall, the difference that was seen is likely precipitated by a wide variety of factors, including the rating distribution in the sample and prescribing patterns.

Another finding was that the average sentiment for high-rated reviews was higher than those of low-rated reviews. Other studies associated exposure to positive sentiment with to a greater likelihood of starting and continuing drug therapy for smoking cessation [6]. This could mean that those who express positive sentiment about a drug are more likely to continue using a medication. This finding is also important because it serves as an important confirmation of the study design. It is expected that the words used in high-rated reviews would be more positive than those used in negative reviews. As such, it would be expected to see the average sentiment be higher in those that rated the medications highly. A person who is rating a medication positively would be more prone to using positive language when reviewing it than a person who rated his or her experience negatively. Even people with a positive experience with a medication will have other experiences that could be included in the review that would lower the sentiment of positive reviews. The review would still be expected to be more positive than negative, however. In the same token, it would be unexpected for a negative review to have significant positive sentiment because the reviewer would be less likely to have other positive experiences that he or she is referencing. 

There was also a difference in average sentiment between SSRIs and SNRIs. The likely explanation of this difference is two-fold. First, the SNRIs had a higher proportion of low-rated reviews in comparison to SSRIs. This would lead to a greater probability that a higher average sentiment review is selected from SSRI reviews than SNRI reviews, which would lead to the difference. The rationale for why SNRIs had lower average sentiment is likely the same as why they had a greater proportion of low-rated reviews. As SSRIs are more often used first-line than SNRIs, it is expected that patients would be more likely to respond to SSRIs than SNRIs as patients receiving SNRIs have more treatment-resistant depression, which would lead to worse results from subsequent therapy.

The limitation to this study is the rule-based lexicon approach. Similar rule-based lexicon approaches using the AFINN lexicon found varying accuracy, ranging from 57.7% up to 63.9% depending on the source of the review [14,19]. Additionally, the AFINN lexicon has been found to be worse at detecting negative sentiment than other lexicons [14,19]. The reduced ability to detect negative sentiment may underestimate the difference between negative sentiment reviews and positive sentiment reviews. Since the AFINN is a general lexicon, it did not include many side effect terms which could be present in medication reviews. Moreover, this study was limited by the reviews, as it was a retrospective analysis of existing reviews. This limited the analysis, as there was no control over the information that was gathered. Other limitations of this study include a manual pull of the medications that were included. Since this was done manually, some medications could have been missed, which could have an impact on the results of the study as well. Sentiment modifiers were captured five words before or two words after and, thus, could result in some modifiers being missed if they occur before or after those limits. If those limits were changed, it would change the results of this study, but it was felt that those limits would reasonably capture the sentiment modifiers.

## 5. Conclusions

This study supports the use of sentiment analysis using the AFINN lexicon, as the lexicon showed a difference in sentiment between high-rated reviews from low-rated reviews. This study also found that SNRIs have more negative sentiment and lower-rated reviews present on Drugs.com^®^ than SSRIs. This could lead patients to have lower expectations when trying an SNRI than an SSRI and, thus, could impact the overall reviews. The order in which medications are tried may also be an underlying factor that is contributing to the difference between SSRIs and SNRIs. More work needs to be done in the area of sentiment analysis and antidepressant reviews, as the overall sentiment of the reviews likely impacts the patient’s willingness to try medications. A future study utilizing these data could be to match SSRI and SNRI reviews based on rating and compare the average sentiment again, to see if there is any deviation in sentiment between the two classes when controlled for rating.

## Figures and Tables

**Table 1:** Demographic information.

	SSRI	SNRI
Number of Reviews	3039	2757
Average Rating for High-Rated	9.19 ± 0.79	9.19 ± 0.79
Average Rating for Low-Rated	1.67 ± 0.83	1.54 ± 0.77
Average Word Count	90.9 ± 46.0	91.1 ± 45.9
Usefulness Count	57.0 ± 76.3	45.1 ± 53.8
Date Range	2010–2017	2011–2017

**Table 2:** Linear model without interaction term.

	Coefficient	SE	Pr
Intercept	−0.372	0.015	<2 × 10^−16^
SSRI	0.012	0.014	0.377
High Rating	0.535	0.016	<2 × 10^−16^

**Table 3:** Linear model with interaction term.

	Coefficient	SE	Pr
Intercept	−0.403	0.019	<2 × 10^−16^
Class SSRI	0.083	0.028	0.003
High Rating	0.578	0.022	<2 × 10^−16^
SSRI: High Rating	−0.093	0.032	0.004

**Figure A1:**
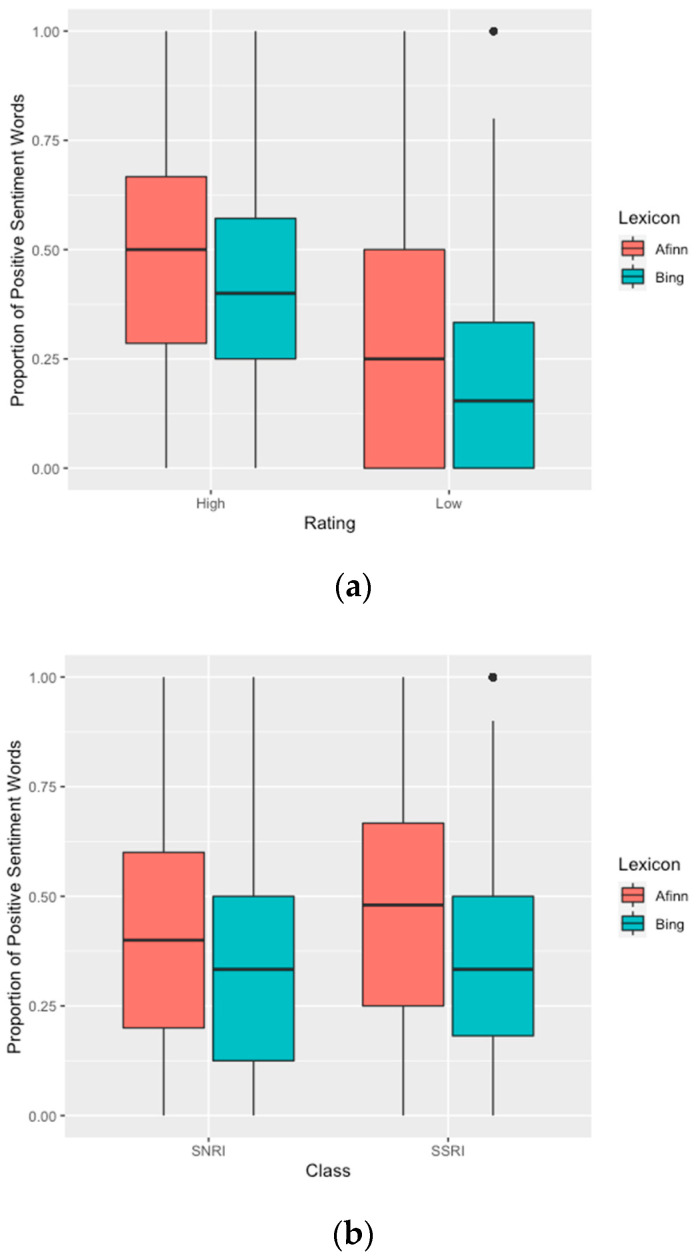
(**a**) This figure shows the distribution of sentiment split by high and low rating, as well as by lexicon. (**b**) This figure shows the distribution of sentiment split between SSRIs and SNRIs, as well as between the BING and AFINN lexicon.

## Data Availability

Publicly available datasets were analyzed in this study. This data can be found here: [https://archive.ics.uci.edu/ml/datasets/Drug+Review+Dataset+%28Drugs.com%29].

## Appendix A

The BING and AFINN lexicons were compared for their ability to analyze the sentiment of reviews. The AFINN lexicon was flattened, to provide an equal comparison between the two lexicons. The lexicons were compared by using the percent of positive sentiment words in the reviews. The graphs above show the differences between the two lexicons. Overall, the AFINN lexicon reported higher sentiment from the reviews, compared to the BING lexicon. The AFINN lexicon also showed a greater variation between the classes of medications. The AFINN appeared to represent a more accurate picture of the information provided in the reviews, based on the fact that the high-rated reviews were only 42% positive sentiment words based on the BING, but 50% positive sentiment words based on the AFINN. Based on this analysis, it was determined that the AFINN lexicon would be better to use for the overall analysis, given the added advantage of giving a scale to the sentiment that is expressed.
